# Inactivation of SAM-Methyltransferase is the Mechanism of Attenuation of a Historic Louse Borne Typhus Vaccine Strain

**DOI:** 10.1371/journal.pone.0113285

**Published:** 2014-11-20

**Authors:** Yan Liu, Bin Wu, George Weinstock, David H. Walker, Xue-jie Yu

**Affiliations:** 1 Department of Microbiology, School of Basic Medical Science, Anhui Medical University, Hefei, Anhui, 230032, China; 2 Department of Pathology, University of Texas Medical Branch, Galveston, Texas, 77555-0609, United States of America; 3 Jiangsu Provincial Center for Diseases Control and Prevention, Nanjing, Jiangsu, China; 4 Department of Genetics, Washington University School of Medicine, St. Louis, MO, 63110, United States of America; 5 School of Public Health, Shandong University, Jinan, Shandong, 250012, China; Kansas State University, United States of America

## Abstract

Louse borne typhus (also called epidemic typhus) was one of man's major scourges, and epidemics of the disease can be reignited when social, economic, or political systems are disrupted. The fear of a bioterrorist attack using the etiologic agent of typhus, *Rickettsia prowazekii*, was a reality. An attenuated typhus vaccine, *R. prowazekii* Madrid E strain, was observed to revert to virulence as demonstrated by isolation of the virulent revertant Evir strain from animals which were inoculated with Madrid E strain. The mechanism of the mutation in *R. prowazekii* that affects the virulence of the vaccine was not known. We sequenced the genome of the virulent revertant Evir strain and compared its genome sequence with the genome sequences of its parental strain, Madrid E. We found that only a single nucleotide in the entire genome was different between the vaccine strain Madrid E and its virulent revertant strain Evir. The mutation is a single nucleotide insertion in the methyltransferase gene (also known as PR028) in the vaccine strain that inactivated the gene. We also confirmed that the vaccine strain E did not cause fever in guinea pigs and the virulent revertant strain Evir caused fever in guinea pigs. We concluded that a single nucleotide insertion in the methyltransferase gene of *R. prowazekii* attenuated the *R. prowazekii* vaccine strain E. This suggested that an irreversible insertion or deletion mutation in the methyl transferase gene of *R. prowazekii* is required for Madrid E to be considered a safe vaccine.

## Introduction

Louse borne typhus (also called epidemic typhus) was one of man's major scourges from the 1500 s through the mid-20^th^ century and frequently played a decisive role in wars in Europe during this period. Thus, it has affected the course of European history [1]. It has killed millions of people, and the threat of louse borne typhus is still real. Louse-borne typhus occurs in epidemics when social, economic, or political systems are disrupted exposing a large population such as refugees to louse infestation due to lack of hygiene and reactivation of latent infection associated with the harsh and stressful conditions. This situation has been observed in outbreaks of typhus in Burundi, Algeria, Peru, and Russia [2]. In 1997, it was estimated that as many as 100,000 cases of typhus occurred in the refugee camps of Burundi [2]. *Rickettsia prowazekii*, the causative agent of louse borne typhus, may be also used deliberately by terrorists in a biologic weapon attack. *Rickettsia prowazekii* is listed as a Select Agent with rigorous legal restrictions on its possession and use in research. The former Soviet Union developed *R. prowazekii* as a biologic weapon during the 1930s [3]. During World War II, the Japanese performed human experiments with rickettsial agents for purposes of biologic weapon development during their occupation of China [4].

Epidemic typhus is transmitted by the human body louse, *Pediculus humanus corporis*. However, the louse is only a vector, but not a reservoir because the louse dies 5–7 days after infection with *R. prowazekii*. *Rickettsia prowazekii* multiplies in louse gut epithelial cells, which detach, rupture and release rickettsiae into the feces and louse hemocoel, killing the insect. The louse feces containing rickettsiae is scratched into the skin, rubbed into mucous membranes such as the conjunctiva, or inhaled. Humans can develop latent infection after acute louse-borne typhus and serve as reservoirs of *R. prowazekii*. *Rickettsia prowazekii* is subsequently reactivated causing recrudescent typhus fever (Brill-Zinsser disease) when latently infected persons experience waning immunity or stressful conditions [5]. *Rickettsia prowazekii* is also maintained in a zoonotic cycle involving flying squirrels (*Glaucomys volans*) and their specific fleas and lice in the eastern United States. Sporadic epidemic typhus occurring in the United States is transmitted to humans by the flea of flying squirrels [6].

Lethality of epidemic typhus in the pre-antibiotic era was usually 15%, and as high as 60%. Even with the availability of effective rickettsiostatic antibiotic treatment, the mortality rate is approximately 4% because of late diagnosis and delay in starting appropriate therapy [2].

After infection, humans develop lifelong immunity to *R. prowazekii* infection or become latently infected. Louse borne typhus is preventable by vaccination using live or dead organisms. The attenuated Madrid E (E) strain of *R. prowazekii*, which had been demonstrated as an effective vaccine, is a spontaneous laboratory variant that was originally isolated from a typhus patient in Madrid in 1941 and passed in rapid succession in embryonated eggs 255 times. The E strain was limited in virulence for guinea pigs and humans. The E strain is protective and provided long term immunity against louse borne typhus when tested as a vaccine during the 1950s and 1960s. Ninety-four percent (170/181) of immunized persons were protected from natural infection by epidemic typhus compared to the unvaccinated controls in a 14-month period after vaccination [7]. Ninety-six percent (27/28) of volunteers who were vaccinated with the E strain and subsequently challenged with Breinl strain at intervals from 2 months to 36 months remained healthy following challenge, and 83% (5/6) of the volunteers who were challenged after 48 to 66 months were protected [8]. However, the E strain vaccine caused a late reaction in up to 14% of vaccinated persons 9–14 days after inoculation. The late reaction varied from simple malaise and mild headache to modified typhus characterized by fever, headache, malaise and occasionally a rash in a small proportion of subjects [7]. The reason for the late reaction was not known at the time. In 1971 a virulent revertant strain (Evir) was isolated by passage of E strain in guinea pigs and/or mice. After the isolation of Evir strain, E and Evir strains have been compared for their biological properties and genetic difference. E strain grows poorly in both human and mouse macrophages, whereas virulent Evir strain and another virulent strain (Breinl) grow very well in these cells [9]. A previous study demonstrated that the patterns of methylation of lysine in the surface protein antigens of E and Evir were different. The surface antigen, outer membrane protein B, of the virulent strains Breinl and Evir is extensively methylated, and that of the avirulent strain E is deficient in protein methylation [10]–[13]. Coincidently, we recently demonstrated that the SAM-methyltransferase gene (*smt*) was inactivated in E strain by a single nucleotide insertion, which was absent in the Evir strain [14]. The important question is whether *smt* is the only gene difference between E strain and Evir strain that causes the reversion of avirulent E strain to virulent Evir strain. To identify the mechanism of attenuation of E strain, we sequenced the genome of Evir strain and compared it to the genome of E strain in the database.

## Materials and Methods

### *Rickettsia prowazekii* strain and genomic DNA preparation

Rickettsiae were cultivated in L929 cell monolayers in 150 cm^2^ flasks. For preparation of genomic DNA, when 100% of L929 cells were heavily infected, cells were disrupted by vortexing vigorously with 4-mm glass beads to release rickettsiae; the cell suspension was centrifuged at 200×g for 10 minutes to remove unbroken cells and nuclei. The supernatant was passed sequentially through a 5 µm-syringe filter and a 3 µm-syringe filter. The filtered solution was centrifuged at 12,100×g for 30 min to collect the rickettsiae. Rickettsial genomic DNA was extracted using the QIAamp DNA Mini Kit (Valencia, CA). In order to determine the TCID_50_, rickettsial stocks were diluted in 10-fold increments from 10^−1^ to 10^−9^. Each dilution was inoculated onto 4 wells of L929 cell monolayers in 6-well plates, and infectivity was determined by examining stained monolayers 12 days later. The TCID_50_ was calculated by the Reed-Muench formula.

### Animal experiments

All experiments were approved by the Institutional Animal Care and Use Committee of the University of Texas Medical Branch (Protocol #: 05-11-074) and conducted in strict compliance with the NIH Guidelines for the Care and Use of Laboratory Animals to minimize any pain or suffering of the animals.

We used guinea pigs to test the virulence of *R. prowazekii* vaccine E strain and virulent strain Evir because guinea pigs are susceptible to *R. prowazekii* infection and develop fever after infection. Other animals except for monkeys are not susceptible to *R. prowazekii* infection. Only animal experiments can determine the virulence of *R. prowazekii* strains, which vary from avirulence to virulence to humans. Therefore, guinea pigs were used in this study. Twenty one male Hartley strain guinea pigs weighing 250 to 300 grams were randomly divided into seven groups with three guinea pigs in each group. The first group was inoculated i.p. with 10^6^ uninfected L929 cells (ATCC CCL-1) in which rickettsiae were cultivated as a negative control. The second, third and fourth groups were inoculated i.p. with Evir strain at doses of 10^4^, 10^5^, and 10^6^ organisms, respectively. The fifth, sixth, and seventh groups were inoculated i.p. with E strain at doses of 10^4^, 10^5^, and 10^6^ organisms, respectively. Body weight and rectal temperature of the guinea pigs were measured daily from the day of inoculation to day 13 after inoculation. On day 14 post infection all guinea pigs were anesthetized by inhaling isofluorane followed by cardiac puncture to collect blood. The guinea pigs were euthanized by using CO_2_ after bleeding.

### Genomic DNA sequencing

The Evir strain was sequenced using the Illumina Genome Analyzer. A subset of reads from which DNA contaminants representing L929 cells and low quality reads had been removed, were de novo assembled into contigs using the Velvet assembler with optimized parameters. The nucmer algorithm (MUMmer package, v3.19) was used to align assembled contigs to the parent Madrid E strain genome and to generate single nucleotide polymorphisms (SNPs) and insertion or deletion (indel) calls. Illumina reads were also mapped directly to the parent genome using BWA, and SNPs were called with Samtools. Variants detected by both programs were further analyzed and validated by PCR for both strains, E and Evir. The Evir strain genome sequence was deposited in GenBank (Access No. PRJNA254815).

## Results

### DNA sequence

Alignment of the genomes of E strain and Evir strain with nucmer revealed that the two genomes were perfectly aligned. There was no large insertion or deletion and no inversion of DNA sequences.

Initial comparison of the genomes of the Evir strain and the E strain (DOW) in GenBank revealed 5 SNPs and 26 indels. We amplified and sequenced the DNA of these SNPs and indels from strain E (DHW) and Evir strain from our laboratory to confirm the SNPs and indels. We found that only one nucleotide was different between Evir strain and E strain (DHW), which was an insertion of a single A at 732 bp in the SAM methyltransferase gene of the strain E (DHW) genome and interrupted the SAM methyltransferase gene near the middle (Table 1). All SNPs and other indels were not present in the strain E (DHW), suggesting that either the genome sequence of strain E(DOW) in GenBank, which was one of the earliest bacterial genomes reported, has errors or the E strain used for genome sequencing was different from E strain (DHW) in our laboratory.

**Table 1:** Mutation in Madrid E strains of *R. prowazekii*.

Strain name	Mutation position/gene name		
	732/*smt*	64/*Rp061*	326/*recO*
Evir			
E (DHW)	T		
E (DOW)	T	T	T

Mutation position was referred to the nucleotide position with the open reading frame of the gene.

To determine whether the SNPs and indels in E strain (DOW) genomic sequence in GenBank were genuine or caused by sequence errors, we acquired a strain E (DOW) that was used for the original *R. prowazekii* genomic sequence from Dr. David Wood's laboratory at the University of South Alabama. We amplified and sequenced all SNPs and indels of strain E (DOW). In addition to the insertion of a T at 732 bp in the SAM methyltransferase gene, strain E (DOW) has two additional nucleotide insertion mutations, which interrupted genes *Rp061* and *recO*, respectively (Table 1). An insertion of a T at position 64 occurred in *Rp061*, which introduced a premature stop codon, resulting in a peptide that lacks the first 19 amino acids. *Rp061* is a conserved protein in *Rickettsia* and is not found in other bacteria. The *Rickettsia recO* encoded a DNA repair protein. The insertion of a T at position 326 of *recO* in *R. prowazekii* also interrupted the gene. Other SNPs which were different between Evir strain and E strain (DOW) in GenBank were not identified in strain E (DHW) and strain (DOW). It suggested that these differences were most likely caused by sequence errors in the GenBank sequence (Accession No: AJ235269.1) (Table 2). The genotype of strain E (DHW) was *smt^−^, recO^+^, rp061^+^* and the genotype of strain E (DOW) was *smt^−^, recO^−^, rp061^−^*.

**Table 2:** Sequence errors in GenBank sequence of strain E (DOW) of *R. prowazekii*.

Position of error	GenBank	Correct sequence	Position	GenBank	Correct sequence	Position	GenBank	Correct sequence
156226		C	570965	C		686469	G	
194784		T	575959		T	695656		C
198098		G	594298	G	A	702501	G	
198987	C	A	617129	G		772338		G
450421	C		620333	.	T	748073	G	
475952		G	621606	C	A	839808		A
594298	G	A	622654	T	C	845044	G	
564697	C		622655	C	A	959787		T
564698	A		646292	A				
570862	C		646476	A				

### Animal experiment

**Figure 1:**
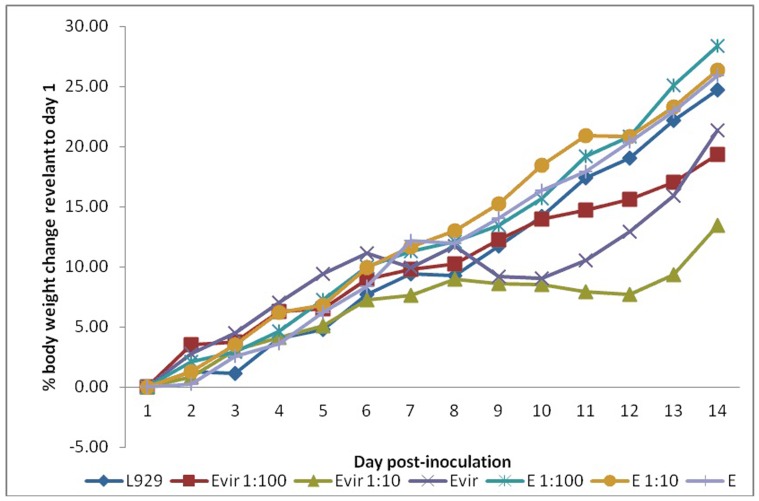
Body weight changes after infection with *R. prowazekii* strain E strain (DHW) and strain Evir at 10^4^, 10^5^, or 10^6^ organisms. L929 cell suspension was used as control.

**Figure 2:**
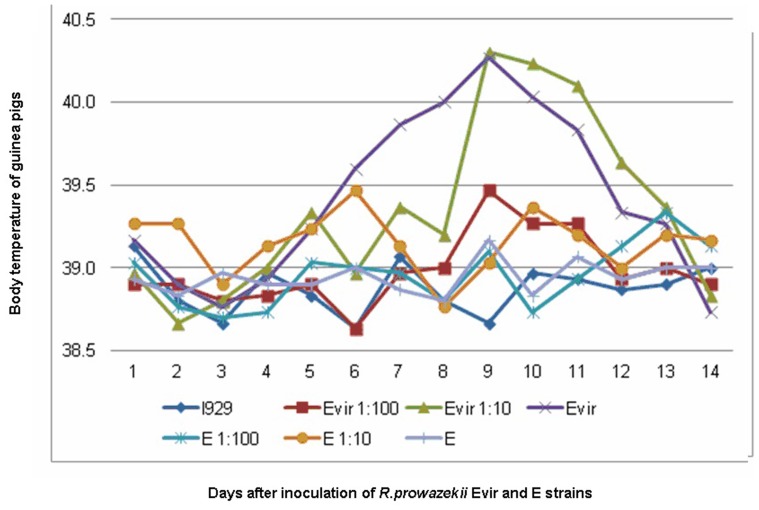
Body temperature changes after infection with *R. prowazekii* strain E (DHW) and strain Evir at 10^6^ (E or Evir), 10^5^ (E1:10 and Evir 1:10), or 10^4^ (E1:100 and Evir 1:100) organisms. L929 cell suspension was used as control.

All guinea pigs inoculated with 10^5^ and 10^6^ organisms of Evir strain developed a fever. After inoculation with a high dose (10^6^ organisms) of Evir strain, one guinea pig had fever from day 5 to day 10, one guinea pig had fever from day 6 to day 11, and one had fever on days 7 and 8. With the medium dose (10^5^ organisms) of Evir strain, all 3 guinea pigs had fever from day 8 to day 11. With the low dose (10^4^ organisms) of Evir strain, only 1 of 3 guinea pigs had a fever from day 8 to 10 after inoculation. The guinea pigs inoculated with the highest dose of Evir strain had a shorter incubation period and a longer period of fever (Figures 1 and 2). In contrast, regardless of the dose (from 10^4^ to 10^6^), no guinea pig inoculated with strain E (DHW) developed fever, and the growth rate of guinea pigs as determined by body weight was not affected by strain E (DHW) infection compared to L929 cell mock-infected guinea pigs. During the febrile period, the growth rate of the guinea pigs inoculated with 10^5^ and 10^6^ organisms of strain Evir was significantly slower than the guinea pigs inoculated with strain E (DHW) or L929 cells. This result confirmed that strain E (DHW) was avirulent and strain Evir was virulent for guinea pigs.

## Discussion

We sequenced the genome of *R. prowazekii* strain Evir and compared it with the genome sequence of strain E (DOW) in GenBank and identified 31 insertions, deletions and SNPs between Evir and E strain (DOW). We sequenced all insertion, deletion and SNPs in strain E (DHW), which we had in our laboratory. We found only one mutation was truly a mutation in strain E (DHW). The mutation interrupted *smt* into two open reading frames, which was designated as rp027 and rp028 previously [15]. We were curious whether the remaining differences between Evir and E (DOW) strain were genuine or caused by sequence errors in the GenBank sequence. We amplified and sequenced all SNPs and indels in a prototype strain E (DOW), which was used for generating the genome sequence of *R. prowazekii* in GenBank (AJ235269.1). Sequence of strain E (DOW) demonstrated that three nucleotides were different between strains Evir and E (DOW), in addition to *smt*, two additional single nucleotide mutations interrupted *recO*, and *rp061* in strain E (DOW). The *recO* and *rp061* mutations have been found in E strain from other laboratory previously [16]. *recO* and *rp061* mutations were not identified in all strains of *R. prowazekii* sequenced.

E strain is avirulent for humans and animals, but passages in animals selected a more virulent strain, Evir. We do not know the origins of strain E (DHW) and strain E (DOW). It is most likely that strain E first acquired the mutation in *smt* and further passages led to accumulation of more mutations such as *recO* and *rp061*.

Mild typhus observed in 14% of vaccinated humans was most likely caused by a mixed population of virulent and avirulent strains. Previously we demonstrated that the methyltransferase gene is inactivated by a frame shift mutation in E strain, but the mutation does not exist in the virulent strain Evir [14]. To determine whether mutation in *smt* determines the attenuation of E strain, we evaluated the virulence of E (DHW) strain in guinea pigs. We demonstrated that E strain (DHW) was avirulent for guinea pigs, but Evir strain is virulent for guinea pigs. After we found that strain E (DHW) and strain E (DOW) were genetically different, we further tested the virulence of E (DOW) in guinea pigs. As expected that E (DOW) also did not cause fever or body weight loss in guinea pigs (data not shown). Since the only three genes different between Evir and E (DOW) and one gene (*smt*) different between strain Evir and strain E (DHW), we reasoned that the reverse mutation in *smt* determined the virulence of *R. prowazekii*. As *smt* encodes a methyltransferase, these results clearly indicate that the methyltransferase gene is critical for the virulence of *R. prowazekii* and the attenuation of E strain might be caused by inactivation of SAM-methyltransferase. It is apparent that SAM-methyltransferase is a virulence factor and deficiency of this enzyme may affect the growth or survival of *Rickettsia* inside the animal host.

A previous study demonstrated that avirulent E strain and Erus had a 12 nucleotide deletion in *rp827*, but all virulent strains including Breinl, Rp22, and Evir had no deletion in *rp827*. In contrast, we found that a 12 nucleotide stretch was deleted from all three strains derived from E strain including Evir, strain E (DOW), and E strain (DHW). It seems highly improbable to have a reverse mutation that inserts 12 nucleotides in *rp827* in Evir strain to restore the mutated gene.

## Conclusions

We concluded that a single nucleotide insertion in the methyltransferase gene *smt* attenuates *R. prowazekii* vaccine strain E, and a reversion mutation makes the revertant strain Evir. It suggests that a safe and irreversible vaccine of *R. prowazekii* can be achieved by genetic deletion of nucleotides of the *smt* gene of *Rickettsia prowazekii* E strain.
